# Brain Metabolism of Allopregnanolone and Isoallopregnanolone in Male Rat Brain

**DOI:** 10.3390/ijms26178559

**Published:** 2025-09-03

**Authors:** Charlotte Öfverman, Martin Hill, Maja Johansson, Torbjörn Bäckström

**Affiliations:** 1 Umeå Neurosteroid Research Center, Department of Clinical Sciences, Umeå University, SE-901 87 Umeå, Sweden; charlotte.ofverman@regionvasterbotten.se (C.Ö.); maja.johansson@diamyd.com (M.J.); 2 Institute of Endocrinology, 110 00 Prague, Czech Republic; mhill@endo.cz

**Keywords:** neuroactive steroids, allopregnanolone, isoallopregnanolone, rat, brain, hippocampus, striatum, circulation, GC-MS, aldoketoreductases, 17β-hydroxysteroid dehydrogenases

## Abstract

Allopregnanolone (allo) and isoallopregnanolone (isoallo) are neuroactive steroid epimers that differ in hydroxyl orientation at carbon three. Allo is a potent GABA-A receptor agonist, while isoallo acts as an antagonist, influencing brain function through their interconversion. Their metabolism varies across brain regions due to enzyme distribution, with AKR1C1–AKR1C3 active in the brain and AKR1C4 restricted to the liver. In rats, AKR1C9 (liver) and AKR1C14 (intestine) perform similar roles. Beyond AKR1Cs, HSD17Bs regulate steroid balance, with HSD17B6 active in the liver, thyroid, and lung, while HSD17B10, a mitochondrial enzyme, influences metabolism in high-energy tissues. Our current data obtained using the GC-MS/MS platform show that allo and isoallo in rats undergo significant metabolic conversion, suggesting a regulatory role in neurosteroid action. High allo levels following isoallo injection indicate brain interconversion, while isoallo clears more slowly from blood and undergoes extensive conjugation. Metabolite patterns differ between brain and plasma—allo injection leads to 5α-DHP and isoallo production, whereas isoallo treatment primarily yields allo. Human plasma contains mostly sulfate/glucuronided steroids (2.4–6% non-sulfate/glucuronided), whereas male rats exhibit much higher free steroid levels (29–56%), likely due to the absence of zona reticularis. These findings highlight tissue-specific enzymatic differences, which may impact neurosteroid regulation and CNS disorders.

## 1. Introduction

**Figure 1:**
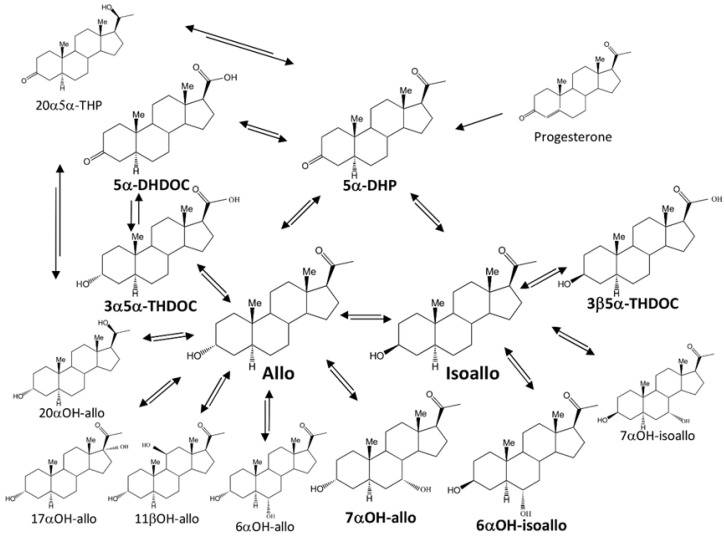
Proposed metabolites and possible metabolic pathways after treatment with allopregnanolone (Allo) or isoallopregnanolone (Isoallo). Steroids analyzed in this study are shown with bold type. Abbreviations: 5α-DHP (5α-pregnane-3,20-dione), allo (3α-OH-5α-pregnane-20-one), 7α-OH-allo (5α-pregnane-3α,7α-diol20-one), isoallo (3β-OH-5α-pregnane-20-one), 6α-OH-isoallo (5α-pregnane-3β,6α-diol-20-one), 5α-DHDOC (21-0H-5α-pregnane-3,20-dione, 5α-dihydrodeoxycorticosterone), 3α5α-THDOC (5α-pregnane-3α,21-diol-20-one, 3α5α-tetrahydrodeoxycorticosterone), and 3β5α-THDOC (5α-pregnane-3β,21-diol-20-one, 3β5α-tetrahydrodeoxycorticosterone). Additional abbreviations for non-analyzed steroids were: 20α5α-THP (20α-OH-5α-pregnane-20-one), 20α-OH-allo (5α-pregnane-3α,20α-diol-20-one), 17α-OH-allo (5α-pregnane-3α,17α-diol-20-one), 11β-OH-allo (5α-pregnane-3α,11β-diol-20-one), 6α-OH-allo (5α-pregnane-3α,6α-diol-20-one), 7α-OH-isoallo (5α-pregnane-3β,7α-diol-20-one). Possible enzymes involved in metabolism: (a) 17β-hydroxysteroid dehydrogenase type 6 (HSD17B6, 3α to 3β-hydroxysteroid epimerase, 3α-3β-HSE) ([12,13], https://www.uniprot.org/uniprotkb/O54753/entry, https://www.uniprot.org/uniprotkb/O14756/entry, accessed on 1 June 2025), (b) 17β-hydroxysteroid dehydrogenase type 10 (HSD17B10) https://www.uniprot.org/uniprotkb/O70351/entry (accessed on 24 June 2025), https://www.uniprot.org/uniprotkb/Q99714/entry, (accessed on 1 June 2025), (c) aldoketoreductase AKR1C9 ([14], https://www.uniprot.org/uniprotkb/P23457/entry, accessed on 1 June 2025), (d) aldoketoreductase AKR1C14 ([15], https://www.uniprot.org/uniprotkb/A0A8I6AD00/entry accessed on 24 June 2025).

Allopregnanolone is a neurosteroid found within neurons throughout the brain [1,2,3], and it has a great impact on CNS function. Allo exerts its rapid effect on the GABA-A receptor, which is part of the main inhibitory system within the brain. The GABA-A receptor is a membrane-bound receptor, consisting of five subunits surrounding a chloride channel. Allopregnanolone (allo, 3α-OH-5α-pregnane-20-one) and isoallopregnanolone (isoallo, 3β-OH-5α-pregnane-20-one) are steroid epimers, structurally differing only in the orientation of the hydroxyl group at carbon three (Figure 1). Both steroids are naturally occurring neuroactive progesterone metabolites that are present in the brain [1,2]. Allo is a strong positive GABA-A receptor agonist [4], and high concentrations are found in blood when the progesterone level is high [5]. Thus, the increased GABA effect of allo inhibits neuronal functions, and thereby allo causes some of the effects seen when the progesterone level is high [6]. Isoallo has by itself no effect on the GABA-A receptor [7,8], but it functions in vivo as an antagonist to allo both in rodents and humans [9,10] and in vitro at specific subunit combinations of the GABA-A receptor [6,8,11].

Recently both allo and isoallo have been used in clinical trials as treatment of postpartum depression concerning allo [16] and treatment of premenstrual dysphoric disorder concerning isoallo [17,18]. In both situations the steroids have been given parenterally to avoid first-passage metabolism in the gut and liver, giving quite high serum levels of the neuroactive steroids in the range of or higher than seen during pregnancy [19,20]. There is, however, the extensive enzymatic capacity in the brain and other parts of the body; therefore, one can expect the presence of multiple metabolites of allo and isoallo in the brain, but as the enzymes are not evenly distributed in the brain, one can expect metabolic variance in different areas [21,22,23,24,25]. It is of interest to know the metabolic fate of allo or isoallo treatments, as some of the metabolites may be neuroactive at both the GABAegic and other receptor systems, like the hormonal receptors. One can expect that some of the metabolites may change the effect of the administered substance or even induce aversive effects. The metabolism of allo and isoallo in the brain has, to our knowledge, not been investigated, and therefore it is difficult to predict possible aversive effects. The aim of this study is to elucidate the main metabolites produced after parenteral administration of maximal dosages of the compounds. A maximal dosage was used to give measurable brain concentrations of the metabolites; at the same time, we wanted to use a dosage that realistically was possible to administer without severe adverse effects.

The choice of brain areas to investigate is due to our interest in allo effects on memory and learning that are related to hippocampus [26], and in previous studies we have noted that the striatum has the highest levels of allo in 9 investigated CNS areas and is therefore a suitable choice. The striatum is a central connection for many areas and is involved in dopamine regulation and substance addiction behavior [27,28,29].

When allo and/or isoallo are produced from progesterone, 5α-dihydroprogesterone (5α-DHP) is formed as an intermediate (Figure 1). The production of 5α-DHP from progesterone is an irreversible reaction [21]. The epimerization of allo into isoallo, and vice versa, can either be with 5α-DHP formed as an intermediate or direct, catalyzed by a single enzyme [12,30,31,32,33].

The synthesis of allo and isoallo steroids occurs in the brain, and in humans the enzymes responsible for the final steps of their synthesis are aldo-keto reductases (AKR1C1–AKR1C4), present in both sexes. Among them, AKR1C1–AKR1C3 are expressed in the brain, whereas AKR1C4 is primarily confined to the liver and is not present in the brain [34,35,36].

Circulating steroid levels and their transport across the blood–brain barrier play a crucial role in regulating steroid concentrations in the central nervous system (CNS) [37,38,39,40]. Therefore, both peripheral and CNS interconversion of steroids may influence CNS steroid levels.

The catalytic activity of AKR1C aldoketoreductases in humans and rats follows a similar pattern, with species-specific isoform variations affecting steroid metabolism. In the reductive direction, AKR1C2 catalyzes the conversion of 3-oxo-5α-steroids to 3α-hydroxy-5α-steroids, while AKR1C4 catalyzes the conversion of 3-oxo-5β-steroids to 3α-hydroxy-5β-steroids. AKR1C1 promotes the conversion of 3-oxo-5α/β-steroids to 3β-hydroxy-5α-steroids, and AKR1C2 also catalyzes the conversion of 3-oxo-5β-steroids to 3β-hydroxy-5β-steroids [36].

Similar to AKR1C2 in humans, the rat AKR1C9 aldoketoreductase is primarily active at the C3 position (https://www.uniprot.org/uniprotkb/P23457/entry, accessed on 1 June 2025). AKR1C9 is primarily expressed in the liver [14]. Besides AKR1C9, another rat aldoketoreductase, AKR1C14, may catalyze the interconversion of 5α-steroids at the C3 position in the reductive direction (https://www.uniprot.org/uniprotkb/P23457/entry, accessed on 1 June 2025). AKR1C14 is predominantly expressed in the small intestine (http://biogps.org/#goto=genereport&id=191574, accessed on 1 June 2025) and is transiently expressed in rat granulosa cells [15].

Beyond AKR1Cs, certain 17β-hydroxysteroid dehydrogenases (HSD17Bs) also regulate the balance between 3α-hydroxy, 3-oxo-5α-hydroxy, and 3β-hydroxy-steroids. HSD17B6 is predominantly found in the human liver, thyroid, and lung (http://biogps.org/#goto=genereport&id=8630, accessed on 1 June 2025) and in the rat small and large intestine (http://biogps.org/#goto=genereport&id=286964, accessed on 1 June 2025). HSD17B6 exhibits broad substrate specificity, performing both oxidation and reduction at the steroid C3 and C17 positions. Additionally, it functions as a 3α/β-hydroxyepimerase (http://biogps.org/#goto=genereport&id=286964, http://biogps.org/#goto=genereport&id=286964, accessed on 1 June 2025). Furthermore, HSD17B10, a mitochondrial enzyme, is involved in fatty acid, branched-chain amino acid, and steroid metabolism and is highly expressed in the human liver (http://biogps.org/#goto=genereport&id=3028, accessed on 1 June 2025). In rats, HSD17B10 is preferentially expressed in high-energy-demand tissues, including the brain, liver, heart, and kidney (http://biogps.org/#goto=genereport&id=63864, accessed on 1 June 2025). HSD17B10’s mitochondrial function is essential for cellular energy metabolism and neurosteroid regulation, catalyzing oxidative steroid conversions at C3, C17, and C20 positions (https://www.uniprot.org/uniprotkb/O70351/entry, https://www.uniprot.org/uniprotkb/Q99714/entry, accessed on 1 June 2025).

In humans, injections of labeled 5α-DHP result in urinary excretion of glucuronidated steroids, mainly 6α-OH-isoallo and 20α-OH-allo, and as a minor product, 6α-OH-allo [41]. In rat tissues, the cytochrome P450 family 7 subfamily B member 1 (CYP7B1) enzyme can catalyze 6α/7α-hydroxylation of 5α-reduced-3β-hydroxy steroids [37,38], a reaction that also occurs in the brain; however, the CYP7B1 does not act on 5β-reduced or 3α-hydroxysteroids [32,41]. In humans, the conversion of progesterone → 5α-DHP → 5α-pregnan-3α/β-ol-20-one → 3α/β,6α-dihydroxy-5α-pregnan-20-one illustrates active extrahepatic steroid metabolism [32,41,42,43].

Ebner et al. in 2006 analyzed the presence of several different possible progesterone metabolites within the naive male rat brain and found no 5β-reduced metabolites [1]. Some of the most common metabolites were 5α-DHP, 5α,20α-THP, allo, and hydroxylated variants of allo (hydroxyl groups in the 11β, 17α, 20α, and 21 positions) [1]. 21-hydroxylated allo is known as tetrahydrodeoxycorticosterone (3α5α-THDOC), a neurosteroid with a strong GABA agonistic effect on the GABA-A receptor like allo. 3α5α-THDOC can either be produced from allo or by reduction of 5α-DHDOC [44].

Metabolite levels are likely influenced not only by the brain region itself but also by the type of steroid administered and the sex of the animal [45]. Only male rats were used to analyze the concentrations of specific steroid metabolites (highlighted in bold in Figure 1), which may be produced from allo and isoallo following treatment with either steroid, and subsequently compared to a control group. [45]. As the formation of 5α-DHP from progesterone is an irreversible reaction, our analyses did not include progesterone and steroids that are direct modifications of progesterone. Neither were 5β-reduced metabolite studies, as the 5β-reductase is not present within the brain [1]. Analyses of steroids were performed in the brain areas hippocampus and striatum. In plasma we also analyzed the sulfate/glucuronided steroids (steroid-C).

The purpose of this study is to elucidate the metabolic fate of exogenously administered allopregnanolone and isoallopregnanolone. The rationale is that they are being clinically administered to humans without the knowledge of the possible presence of neuroactive compounds in the brain and thereby reasons for possible aversive effects and interactions with endogenous steroid actions. This information does not exist in scientific literature, and this information was not published earlier.

## 2. Results

For analyses of the steroid metabolites, a gradient enabling complete separation of steroid isomers from each other and from possible interfering substances was used. As shown in Table 1, separation of steroid isomers with almost identical fragmentation was accomplished. We also considered fragmentation patterns and physiological concentrations of steroids and sterols, which can be co-eluted with the measured substances, and found no critical interference.

### 2.1. Method Development for Steroid Analyses

The correct identifications of the substances were ensured by congruence of fragmentation pattern and retention time with the used standards (at least 2 fragments + retention time). Further, most steroids containing an oxo-group produced a two-peak response; in these cases, we also controlled the ratios of peak 1 to peak 2 for individual fragments.

### 2.2. Steroid Concentrations in Vehicle Treated Animals

In the plasma of the control animals, there were measurable amounts of all steroids analyzed (Table 2). The highest concentrations were found for allo, followed by 5α-DHP, 3α5α-THDOC and 5α-DHDOC, isoallo, and lower amounts of the rest of the analyzed steroids. The plasma allo concentration was 16.5 times higher than 5α-DHP but 264 times higher than isoallo. In the presentation of the results below, the concentration ratios are displayed. The reason is to give a sense of the concentration differences and degree of metabolism that occurs in the control animals.

In the hippocampus, of the vehicle-treated group, allo showed the highest concentration, followed by 5α-DHDOC, isoallo, and 5α-DHP. The concentrations are thus different from the plasma concentrations (Table 2 and Table 3). The ratio between the steroids was different in the hippocampus compared to plasma, as allo was only 7.17 times higher than isoallo, thus quite different from plasma. 5α-DHP, however, showed a similar ratio to allo, namely 14.9 times. This indicates that in the hippocampus there is, in controls, a higher isomerization between allo and isoallo or new production of isoallo compared to plasma.

In the striatum the allo concentration was lower than in the hippocampus, and the pattern was slightly different, as isoallo was higher than 5α-DHDOC and 5α-DHP was quite low (Table 4). The ratio between allo and 5α-DHP was 23.0, while it was 4.79 for isoallo.

Sulfate/glucuronided steroids did not exactly follow the same pattern; allo-C had the highest concentration, followed by 5α-DHDOC-C, isoallo-C, and 6α-OH-isoallo-C. Allo was in the analyzed brain areas, hippocampus and striatum, dominating, followed by isoallo or 5α-DHDOC, 5α-DHP, 3β5α/3α5α-THDOC, and 6α-OH-isoallo (Table 3 and Table 4).

### 2.3. Steroid Concentrations in Allo Treated Animals

In plasma, animals treated with allo (2 mg/kg) showed significantly higher levels of the studied steroids, except for 5α-DHDOC, isoallo, and the two hydroxylated isoallo metabolites (3β5α-THDOC and 6α-OH-isoallo, Table 2) compared to controls. The concentration ratio allo/5α-DHP showed a 20.5-fold difference, and the ratio allo/isoallo showed a 427-fold difference. In the isoallo-treated group, the concentration ratio isoallo/allo is 23, and for isoallo/5α-DHP = 85. The ratio suggests that the isomerization in plasma between allo and isoallo is not high in male rats, while the isomerization from isoallo to allo is much higher (Table 2).

Among the sulfate/glucuronided steroids in plasma, only allo-C was significantly increased compared to the amounts found in control animals. The concentrations of sulfate/glucuronided steroids were generally similar to those of their non-sulfate/glucuronided counterparts, except for 5α-DHDOC-C, which exhibited higher levels than non-sulfate/glucuronided 5α-DHDOC. In the hippocampus the concentrations of allo, isoallo, and 5α-DHP were significantly higher than in the control group (Table 3). The ratio of allo/5α-DHP was 35.2, and for allo/isoallo, 55.1. In the striatum only allo and 5α-DHP had significantly higher levels compared to the control (Table 4). The ratio of allo/5α-DHP was 53.8 times, and the ratio of allo/isoallo was 72.9 times. Thus, the conversion of allo to 5α-DHP seems to be in the same range in the hippocampus and striatum as in plasma, but the isomerization of allo to isoallo appears to be higher in the hippocampus and striatum than in plasma. In both the hippocampus and striatum, a similar concentration of 5α-DHP was found compared to plasma: 85.0 nmol/kg and 37.3 nmol/kg vs. 35.0 nmol/L. However, the concentration of isoallo after allo treatment was different in the hippocampus and striatum: 54.2 nmol/kg and 27.5 nmol/kg compared to plasma vs. 1.6 nmol/L. There was thus a higher concentration of isoallo after allo treatment in the hippocampus and striatum compared to plasma.

### 2.4. Steroid Concentrations in Isoallo Treated Animals

In the animals given isoallo resulted in increased levels of all the studied steroids in plasma (Table 2) and all but one of the studied sulfate/glucuronided steroids (5α-DHDOC-C), compared to the controls. In plasma the isoallo/allo ratio was 22.9, and isoallo/5α-DHP was 84.6 times.

In the hippocampus, all steroids were increased compared to the control levels (Table 3). The isoallo/allo ratio was 60.8, and for isoallo/5α-DHP, 520 times.

In the striatum, all steroids but 7α-OH-allo were significantly changed compared to controls, but the changes were non-significant after the Bonferroni adjustment (Table 4). The isoallo/allo ratio was 74, and isoallo/5α-DHP was 389 times. It seems thus that the conversion of isoallo to 5α-DHP is low in the hippocampus and striatum while, the isoallo-to-allo conversion seems similar as in plasma.

### 2.5. Adjusted Changes of Metabolites

**Figure 2:**
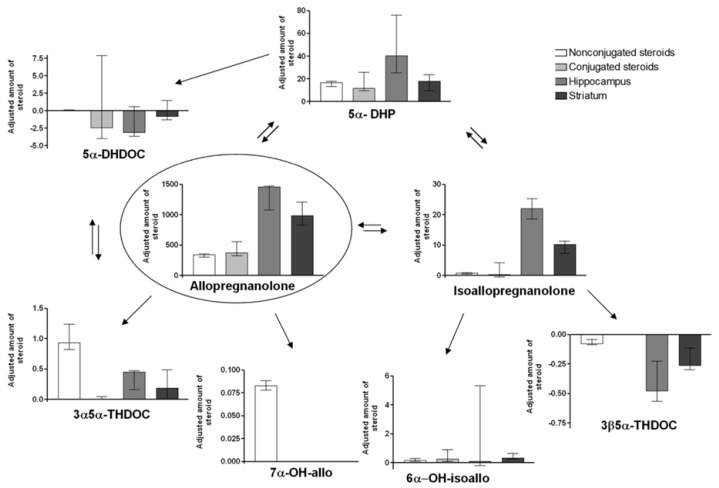
This figure shows the major metabolites after parenteral administration of allopregnanolone. Relative concentration changes (nmol/L or nmol/kg) in the amounts of steroid metabolites are normalized, compared to control concentration = 0, adjusted for the dosage given, in this case 2 mg allo/kg (median ± 25–75 percentiles). The adjusted concentration changes were calculated by dividing the change in metabolite concentration for each tissue with the amount of steroid given. That is; for the allo treated group [metabolite]_treated_ − [metabolite]_control_/2 mg/kg allo given. Non-sulfate/glucuronided steroid in plasma is shown in white column, sulfate/glucuronided metabolites in light gray, Hippocampus concentration changes in dark gray and striatum concentration change in black columns. Significant changes are described within the text. Abbreviations of steroid names as described in Figure 1 and the Material and Method Section 4.1, Chemicals.

**Figure 3:**
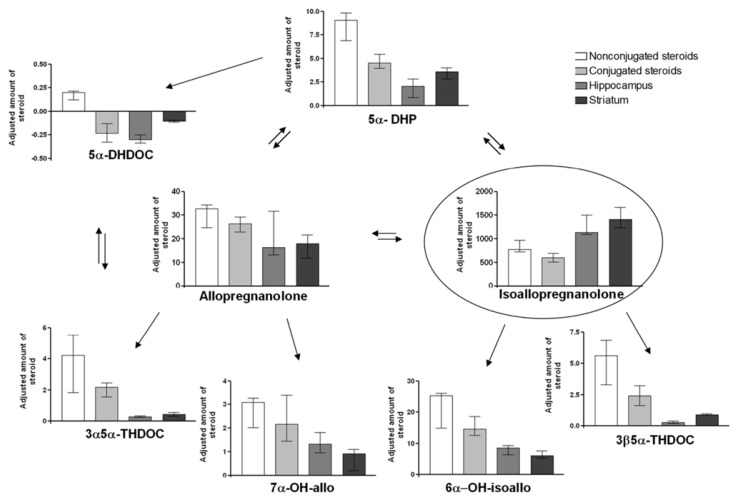
This figure shows the relative concentration of the major metabolites after parenteral administration of isoallopregnanolone. Relative concentration changes (nmol/L or nmol/kg) in the amounts of steroid metabolites, compared to control concentration = 0, adjusted for the dosage given, in this case 32 mg isoallo/kg (median ± 25–75 percentiles). The adjusted concentration changes were calculated by dividing the change in metabolite concentration for each tissue with the amount of steroid given. That is; for the isoallo treated group [metabolite]_treated_ − [metabolite]_control_/32 mg/kg isoallo given. Nonsulfate/glucuronided steroid in plasma is shown in white columns, sulfate/glucuronided metabolites in light gray, Hippocampus concentration change in dark gray and striatum concentration change in black columns. Significant changes are described within the text. Abbreviations as described in Figure 1 and the Material and Method Section 4.1, Chemicals.

For comparisons of the amounts of steroid metabolites formed after the different treatments, the increase/decrease compared to control was calculated, and this delta value was divided by the amount of given steroid in mg/kg, i.e., either allo 2 mg/kg or isoallo 32 mg/kg. The diagrams for the different metabolites and tissues analyzed are shown in Figure 2 (allo treatment) and Figure 3 (isoallo treatment).

Allo treatment: As seen in Figure 2, the highest adjusted steroid amount was found for allo, which had accumulated within the brain (*p* ≤ 0.001 for change in hippocampus or striatum vs. steroid in plasma). The main metabolite formed was 5α-DHP, followed by isoallo. Both these steroids also had accumulated in the brain; this was especially pronounced for isoallo, with a hippocampal increase about 20 times higher than in plasma (*p* ≤ 0.001 for change in hippocampus or striatum vs. non-sulfate/glucuronided steroid in plasma). On the contrary, the formed 7α-OH-allo was only present within the blood, and 3α5α-THDOC was also significantly higher in the blood compared to within the brain tissue (*p* ≤ 0.001 for hippocampus and *p* ≤ 0.05 for striatum).

Isoallo treatment: Figure 3 shows that the pattern was slightly different when isoallo was given. The highest adjusted change was found for the given steroid isoallo, while the accumulation within the brain was not as pronounced as for allo. Instead, the isoallo was more evenly distributed between blood and the brain areas, but still the amount of isoallo was higher in the brain than in plasma (*p* ≤ 0.01 for hippocampus or striatum vs. non-sulfate/glucuronided steroid in plasma). Interestingly, the main metabolite found after the isoallo injection was allo, but the formed allo was not accumulated within the brain (NS). The lack of brain accumulation was also true for the rest of the metabolites formed after the isoallo injection. The steroid changes in plasma were instead dominating. After the isoallo injection, the second most common metabolite formed was 6α-OH-isoallo, followed by 5α-DHP. Low amounts were also formed of the rest of the steroids studied.

Statistical comparisons of the data in Figure 2 and Figure 3 revealed differences in the adjusted increases of the injected steroids. Isoallo levels were significantly higher following isoallo injection in both the striatum and plasma, and isoallo-C levels were also elevated in plasma, compared to the adjusted increases of allo in the same tissues following allo injection. The degree of epimerization between allo and isoallo varied between the injections: isoallo injection produced higher adjusted changes of allo in the striatum and plasma, and allo-C in plasma, than the adjusted changes in isoallo and isoallo-C observed after allo injection. These results suggest that isoallo is more readily taken up by the brain than allo.

Significant differences were also observed in the adjusted levels of metabolites formed following the two types of injections. The only metabolite found in higher concentrations after allo injection compared to isoallo injection was 5α-DHP, particularly in the hippocampal tissue. In contrast, isoallo injection resulted in significantly higher levels of 7α-OH-allo, 6α-OH-isoallo, and 3β5α-THDOC than were observed after allo injection.

## 3. Discussion

Activation with positive GABA-A receptor modulating neurosteroids and/or potentiation of the neurotransmitter GABA at the receptor both lead to prolonged opening of the chloride channel that normally gives an influx of chloride ions and decreased membrane potential. A concentration in plasma of allo in the range of 80–200 nmol/l leads to sedation [16,46,47,48,49], decreased learning [46], increased disease deterioration in Alzheimer mice [10], and a high concentration up to 530–1700 nmol/l leads to anesthesia [47,48]. The physiological concentration at the end of pregnancy is up to 100 nmol/L [48,49]. With such high impact, it is probable that the concentration of allo within the brain is tightly regulated. This could be achieved at several levels, i.e., controlled uptake and/or excretion, and by regulation of the synthesis as well as the degradation.

In our study the concentration in control plasma after vehicle administration showed a large difference between especially the isoallo and allo concentrations, with 264 times higher levels of allo compared to isoallo. The allo concentration in the present study was somewhat lower than in the study by [50,51] in the hypothalamus and [52] in the hippocampus of male rats.

The isoallo level was not detectable in their assay, suggesting a similar relation between allo and isoallo as in the present study. In women during the follicular phase, the allo concentration is nearly doubled compared to isoallo, with the same difference during the luteal phase [5]. This suggests that the peripheral isoallo concentration/production in male rats is different from humans. In the hippocampus and striatum, the isoallo concentration was much closer to the allo concentration (7 and 5 times), suggesting that the 3β-hydroxylation has a higher capacity in the brain areas than in the periphery.

We know from earlier studies using allo both in rodents and humans that the effect of allo is very rapid. We can note the effect on brain activity at the time when the neuroactive steroid reaches the brain tissue [53]. The common route for steroid metabolism is hydroxylation, to make the compound more water soluble, or sulfation by sulfotransferases or by conjugation with glucuronic acid before excretion.

The metabolic pathways of allo and isoallo following parenteral administration remain unknown and have not been previously studied; however, it is plausible that their metabolism involves the enzymatic systems naturally present in the brain [21,54]. Therefore, we give here a short resume. The enzymes involved in the last steps of the allopregnanolone and isoallopregnanolone synthesis and metabolism in humans are aldo-keto reductases (AKR1C1–AKR1C4) in both sexes. AKR1C1–AKR1C3 are expressed in, among other organs, the brain, while AKR1C4 (3α-hydroxysteroid dehydrogenase, HSD) is largely confined to the liver and is not present in the brain [35,36,55]. There is evidence that in vivo all isoforms will predominantly work as reductases [35,36].

Under normal conditions in humans, AKR1C1 predominantly reduces hormonal steroids to their inactive metabolites, particularly progesterone to 20α-dihydroprogesterone. However, AKR1C1 is also the main enzyme producing the 3β metabolites of 5α-reduced steroids [55], and in vivo AKR1C1 favors the formation of 3β-OH compared to 3α. In the central nervous system, the AKR1C1 must be considered the source for the formation of 3β-tetrahydrosteroids [34,55]. AKR1C2 metabolizes 5α-DHP to 3α5α-pregnanolone (allopregnanolone) [56,57]. AKR1C2 acts as an efficient and almost exclusive 3α-reductase. AKR1C3 (3α-Hydroxysteroid dehydrogenase; 17β-hydroxysteroid dehydrogenase) acts mainly as a 17-ketosteroid reductase but also has a weak 3α(3β)-reductase capacity in the brain [36]. AKR1C4 is an efficient 3α-HSD with subsidiary 3β-HSD activity but mainly acts in the liver, not in the brain. In vivo AKR1C1 is considered the only enzyme for the 3β-tetrahydrosteroid formation within the central nervous system. AKR1C2 is believed to be responsible for the formation of the inhibitory 3α-OH-steroids that are positive GABA-A receptor modulating neurosteroids like allo, pregnanolone, and allotetrahydrodeoxycorticosterone (THDOC) [34,35,36,55,56,58,59,60,61]. A hyper function of AKR1C2 may lead to an overproduction of allopregnanolone, which may result in increased sedation [62]. An overactive AKR1C2, due to increased expression, has been shown in an individual suffering from severe chronic fatigue [63].

Unlike in humans, in rats, the balance between 3α-hydroxy, 3-oxo-5α-hydroxy, and 3β-hydroxy-steroids is influenced by AKR1C9 (https://www.uniprot.org/uniprotkb/P23457/entry, accessed on 1 June 2025) and AKR1C14 (https://www.uniprot.org/uniprotkb/A0A8I6AD00/entry, accessed on 1 June 2025), while HSD17B6 (https://www.uniprot.org/uniprotkb/O70351/entry (accessed on 1 June 2025), and https://www.uniprot.org/uniprotkb/O14756/entry, accessed on 1 June 2025), and HSD17B10 (https://www.uniprot.org/uniprotkb/O70351/entry (accessed on 1 June 2025) and https://www.uniprot.org/uniprotkb/Q99714/entry, accessed on 1 June 2025) contribute to this balance in both species.

Eight minutes after intravenous administration of allo, the highest steroid concentration detected in the brain was from the unmetabolized compound, indicating its accumulation in brain tissue. In circulation, both sulfate/glucuronided and non-sulfate/glucuronided forms of allo were present at similarly high levels. This pattern suggests that rapid conjugation of the unmetabolized steroid occurred in peripheral tissues. Notably, no sulfate/glucuronided forms were detected in the brain.

The main allo metabolites formed, 5α-DHP and isoallo, might represent the first steps in the degradative pathway of allo, as a mandatory formation of isoallo has been proposed for allo degradation in the CNS [32]. In Stromstedt’s study no polar allo metabolites were found after incubation with microsomes from rat brain [32]. In this study we found 7α-OH-allo in plasma but not in the brain, but 3α5α-THDOC (21-hydroxylated allo) both in plasma and brain. Several other hydroxylated metabolites of allo might have been produced but were not analyzed. The two main allo metabolites, isoallo and 5α-DHP, are both without positive effects on the GABA-A receptor; on the contrary, isoallo can, at least at certain variants of the receptor, function as an allo antagonist [6,8,11], i.e., a relief of the GABA-A receptor activation started by the production of these metabolites. With 5α-DHP as a main metabolic outcome, one can suspect that the ARK1C2 (3α-HSD) can act in both directions as discussed above. The 3α5α-THDOC was rather high in the brain and plasma, suggesting the 21-steroid hydroxylase exists both in the brain and in the periphery.

For all the allo metabolites formed, high concentrations were found in the brain, and they might then have been formed within the brain. This is in accordance with the study by Vallée et al. 2000, where the subcutaneous injected allo twenty minutes after the injection was found in plasma and in the brain, while the formed isoallo only was present in the brain [33]. This might then indicate that allo is converted into isoallo predominantly within the brain. This hypothesis is supported by our findings, as the plasma concentrations of isoallo were low after allo IV injection.

According to the literature, the epimerization between allo and isoallo can either be catalyzed by a 3α to 3β-hydroxysteroid epimerase (3α-3β-HSE) preferring NAD and NADH as cofactors, different from the AKR1C1-C4 preferring NADPH as a cofactor [12,30,64]. A second possible epimerization takes place in two separate enzymatic steps with 5α-DHP formed as an intermediate [31,32,33]. The enzymes needed for both epimerization routes have been reported to be present in the brain [12,30,32]. The high amount of 5α-DHP accumulated after the allo injection suggests the usage of the latter route when allo is the injected steroid.

When isoallo was injected, an accumulation of isoallo within the brain was also seen, with similar amounts of steroid present per injected amount as for allo. However, the adjusted change in isoallo was higher in plasma than for allo, indicating that isoallo requires more time for clearance from the blood compared to allo. Also, a high level of isoallo conjugation was found. There is then a remarkable capacity for quick conjugation of steroids, considering the large amount of sulfate/glucuronided isoallo present eight minutes after the IV injection (about 20 µmol/L). In plasma the dominating metabolites after the isoallo injection were allo, followed by 6α-OH-isoallo and 5α-DHP. It is interesting that these metabolites were not mainly found in the brain. Thus, either the metabolites were formed outside the brain, or they were produced within the brain and then transferred out to the blood stream. A possible way to pursue answering this question would be to follow metabolism over several time points. In the present study only a single time point was studied.

The main metabolite formed after the isoallo injection was its epimer allo; this is contrary to what was found after the allo injection, where 5α-DHP was the main metabolite. The 3α to 3β-hydroxysteroid epimerase enzyme studied by Huang et al. showed low activity in the 3β- to 3α-OH direction [12,13], making it less probable that this enzyme is responsible for the epimerization from isoallo into allo. Other enzymes shown to possess this epimerase activity have not been studied for activity in the 3β- to 3α-OH direction [30,64]. On the other hand, the adjusted change of the intermediate 5α-DHP, formed during the two-step interconversion, was not high. This could anyhow be the actual route and might indicate that there is a high capacity for conversion of 5α-DHP into allo, as the 5α-DHP concentration was relatively low. 6α-OH-isoallo seems to be an important metabolite, as this was the main metabolite secreted in the human urine after treatment with labeled 5α-DHP [41] and was also identified as one of the metabolites formed after isoallo incubation with brain microsomes [32].

There was an overload of steroids used in this study, the reason being to be able to identify and measure low-capacity metabolites after the injection of isoallo. This overload might have influenced the preferred metabolic pathways compared to the situation with physiological concentrations. The concentration after parenteral administration in the present study was higher than the concentration of isoallo obtained in the human clinical trial with isoallo after sc. injections [18]. The higher adjusted changes of steroids in the blood after treatment with the higher dose of steroid, 32 mg isoallo/kg compared with 2 mg allo/kg, might also point to a regulated uptake into the brain or the possibility of depletion of steroids from the brain when the blood concentration is too high.

In the study by Vallée et al., the amount of isoallo formed from the injected allo was roughly around 5% of the allo concentration in the frontal cortex of the rat [33]. This analysis was performed 20 min after the s.c. injection of allo, and there was no detectable isoallo in plasma. On the contrary, we could quantify isoallo in both the brain and plasma after the IV injection of allo, with the amounts of isoallo being around 1–4% of the concentrations of allo. The same was true for the isoallo treatment, with the concentrations of allo then being 0.2–1.5% of the isoallo concentrations. It should then be remembered that a large dose of isoallo was used, so the low percent of conversion resulted in a high concentration of allo. In the present study the steroid concentrations were analyzed at only one time point, making it impossible to know if the amounts of epimers measured are increasing, decreasing, or at the highest concentrations possible to reach after the injections. In women, Hedström et al. showed that IV injection of isoallo results in formation of allo, and the concentration of produced allo found in serum was 10–15% of the isoallo concentration at the different time points [65]. In in vitro experiments where human placenta tissue was incubated with isoallo for 120 min, 5α-DHP and allo were the steroids found to accumulate, while very little isoallo remained at the end of the incubation [31]. While an identical experiment, but with allo as the original steroid, showed that 5α-DHP, allo, and isoallo were produced at quite even concentrations [31]. Thus, the epimerization route and/or enzyme activities needed for the conversions between allo and isoallo seem to be different in different tissues.

In healthy women both allo and isoallo are in the circulation, mainly found as sulfate/glucuronided, with only 2.4–6% found as non-sulfate/glucuronided [5]. This was true in both the follicular and the luteal phases. This is then contrary to the situation here found in the male rat, especially after the treatments with steroids, where 29–56% of allo and isoallo in plasma were non-sulfate/glucuronided. This finding could be related to the absence of zona reticularis in rats. In humans, this zone exhibits extreme sulfotransferase activity [66,67]. In many studies the source of the sulfate/glucuronide has not been determined; it was not done in this study either. Both glucuronide and sulphate (sulfate/glucuronides) have been reported and there are indications that the 3β-position is preferentially sulphated [68]. On the other hand, after treatment of both men and women with labeled 5α-DHP, the main metabolite excreted in the urine was glucuronidated 6α-OH-isoallo (i.e., a 3β-hydroxy steroid) [41]. However, the conjugation might, in this case, be in the 6α-position.

### 3.1. Potential Clinical Implications of the Findings

As isoallo behaves as an allopregnanolone antagonist, it is of interest that a large amount of allopregnanolone is converted to isoallo. The case might be that interconversion between allo and isoallo is a mechanism for the brain to achieve control of over activity in the GABA-A receptor system and thus inhibition, especially in disorders with fatigue and decreased cognitive symptoms.

### 3.2. Future Directions

It would be interesting to do a dose–concentration time curve and to study other species, especially primates, especially dose–effect curves, perhaps via imaging and MRI studies during treatments.

### 3.3. Limitations and Strengths of the Study

One limitation is that this study is made in rats, and rodents have different enzyme systems that might be of importance for steroid metabolism. A second is that only one time point is used, and rather few animals were used. The strength is that the assays are robust and trustworthy. As the loading dosage was high, one can expect that the major metabolic pathways were discovered.

## 4. Materials and Methods

### 4.1. Chemicals

Drugs given to the animals were allopregnanolone (allo, 3α-OH-5α-pregnane-20-one) 2 mg/mL (Umecrine AB, Umea, Sweden) and isoallopregnanolone (isoallo) 3 mg/mL (Sigma-Aldrich, St Louis, MO, USA). Both steroids were dissolved in 10% 2-hydroxypropyl-β-cyclodextrin (Sigma Chemicals Co, St. Louis, MO, USA) using ultrasound.

During GC-MS analysis, the following steroids from Steraloids (Newport, RI, USA) were used as standards: 5α-DHP (5α-pregnane-3,20-dione), allo (3α-OH-5α-pregnane-20-one), 7α-OH-allo (5α-pregnane-3α,7α-diol20-one), isoallo (3β-OH-5α-pregnane-20-one), 6α-OH-isoallo (5α-pregnane-3β,6α-diol-20-one), 5α-DHDOC (21-0H-5α-pregnane-3,20-dione, 5α-dihydrodeoxycorticosterone), 3α5α-THDOC (5α-pregnane-3α,21-diol-20-one, 3α5α-tetrahydrodeoxycorticosterone), and 3β5α-THDOC (5α-pregnane-3β,21-diol-20-one, 3β5α-tetrahydrodeoxycorticosterone). The Sylon B used was from Supelco (Bellefonte, PA, USA), methoxylamine hydrochloride from Sigma (St. Louis, MO, USA), and the solvents from Merck (Darmstadt, Germany).

Additional abbreviations used in Figure 1 for non-analyzed steroids were 20α5α-THP (20α-OH-5α-pregnane-20-one), 20α-OH-allo (5α-pregnane-3α,20α-diol-20-one), 17α-OH-allo (5α-pregnane-3α,17α-diol-20-one), 11β-OH-allo (5α-pregnane-3α,11β-diol-20-one), 6α-OH-allo (5α-pregnane-3α,6α-diol-20-one), and 7α-OH-isoallo (5α-pregnane-3β,7α-diol-20-one).

### 4.2. Animal Studies

Adult male Wistar rats (300–365 g, n = 16, Taconics, Sikeborg, Denmark) were treated IV in the tail with allo (2 mg/kg, n = 7), isoallo (32 mg/kg, n = 5), or 2-hydroxypropyl-β-cyclodextrin (1 mL/kg, n = 4). The 2 mg/kg allo dosage was used as a higher dosage that caused anesthesia and influenced oxygenation. The dosage of isoallo up to 32 mg/kg gave no signs of toxic effect and was therefore possible to use. Eight minutes after the injections, the rats were decapitated, trunk blood was collected, and the brain was immediately removed from the skull and, while kept on ice, dissected into specific brain areas. The animals used in the study had, before this injection, been given the same living conditions as in earlier studies [46]. The tissue was weighted and either frozen on dry ice (for later use) or directly extracted in ethanol. Before shipping for analysis, the tissues kept in ethanol were dried. This was done due to shipping regulations; further processing was described below. The blood samples were centrifuged, and the collected plasma was stored in −20 °C.

Treatment of the animals was in accordance with the European and Swedish legislation, and the study was approved by the Regional Ethical Committee for Animal Research in Umea, Sweden (approval code: A103; approval date: 25 October 2002).

### 4.3. Instruments/Method

The GC-MS analyses have partly been described earlier [19]. The GCMS-QP2010 Plus system from Shimadzu (Kyoto, Japan) consisted of a gas chromatograph equipped with an automatic flow control, an AOC-20s autosampler, and a single quadrupole detector with an adjustable electron voltage from 10 to 195 V. A capillary column with a medium polarity REST EK Rxi (diameter 0.25 mm, length 15 m, film thickness 0.1 pm) was used for analyses. Electron-impact ionization, with electron voltage fixed at 70 V and emission current set to 160 PA, was used for the measurements. The temperatures of the injection port, ion source, and interface were maintained at 220 °C, 300 °C, and 310 °C, respectively. Analyses were carried out in the splitless mode with a constant linear velocity of 60 cm/s for the carrier gas (He). The septum purge flow was set to 3 mL/min. The samples were injected using the high-pressure mode, 200 kPa, which was maintained for 1 min. The detector voltage was set to 1.4 kV. The temperature gradient used was an increase from 80 to 210 °C (40 °C/min), an increase to 215 °C (0.6 °C/min), an increase to 300 °C (20 °C/min), and a 5 min delay at 300 °C. The initial pressure was 34 kPa, the injector temperature 220 °C, and the analysis duration 20.83 min.

### 4.4. Sample Pre-Treatment

The plasma samples were processed as follows: the original samples and the polar phases after diethyl-ether extraction (see below), which were used for the quantification of the steroid sulfate/glucuronides, were spiked with 17α-estradiol (as an internal standard) to attain a concentration of 1 ng/mL and 10 ng/mL, respectively. The unsulfate/glucuronided steroids were extracted from 1 mL of plasma with 3 mL of diethyl-ether. The diethyl-ether extract was dried in a block heater at 37 °C. The lipids of the diethyl-ether extract were separated by partitioning between a mixture of methanol-water 4:1 (1 mL) and pentane (1 mL). The pentane phase was discarded, and the polar phase was dried in a vacuum centrifuge at 60 °C (2 h). The dry residue from the polar phase was first derivatized with methoxylamine-hydrochloride solution in pyridine (2%) on oxo-groups (60 °C, 1 h). The mixture after the first derivatization was dried in the flow of nitrogen, and the dry residue was treated with the reagent Sylon B (99% of bis (trimethylsilyl) trifluoroacetamide and 1% of trimethylchlorosilane), forming trimethylsilyl derivatives on hydroxy groups (TMS-MOX derivatives, 90 °C, 1 h). Finally, the mixture after the second derivatization step was dried in the flow of nitrogen, the dry residue was dissolved in 20 µL of isooctane, and 1 µL of the solution was used for GC-MS analysis.

Steroid sulfate/glucuronides remaining in the polar phase after the diethyl-ether extraction were analyzed as follows: The polar residues were dried in a vacuum centrifuge at 37 °C (5 h) and then hydrolyzed as described elsewhere [69]. The hydrolyzed samples were again dried in a vacuum centrifuge at 37 °C (5 h) and then reconstituted with 1 mL of chromatographic water and processed in the same way as the free steroids (described above). In contrast to the sample preparation of free steroids, the dry residue after the second derivatization step was dissolved in 200 µL of isooctane.

The tissue samples were processed as follows. Ethanol (1 mL) was added per 15 mg of tissue (rat hippocampus or striatum), and the sample was incubated at 4 °C for 20 h. The ethanol extract was dried in a vacuum centrifuge at 40 °C. After that, the aliquot of internal standard, 500 µL of water, and 3 mL of diethylether were added, and the sample was extracted for 2 min. The diethyl-ether extract was further processed in the same way as in the case of unsulfate/glucuronided steroids in plasma.

The internal standard was recorded at effective masses *m*/*z* = 231, 285, and 416. The addition of an internal standard to the plasma before sample preparation (free unsulfate/glucuronided steroids) and to the polar phase after diethyl-ether extraction (sulfate/glucuronided steroids) assured that losses during sample processing were not critical for steroid quantification. The addition of an appropriate steroid sulfate/glucuronide as the internal standard into the original sample would have been more correct, but we were unable to find an appropriate commercially available steroid sulfate/glucuronide. The efficiency of the hydrolysis step was verified for several commercially available steroid sulfate/glucuronides (steroid-C), comparing known quantities of unsulfate/glucuronided steroids (1 ng) after diethyl-ether extraction with equimolar amounts of corresponding sulfates after hydrolysis and diethyl-ether extraction of the released steroids. The efficiency of the hydrolysis was tested in 10 parallel measurements for steroid sulfates with the following results: 98.4 ± 1.8% (androsterone), 89.8 ± 1.3% (etiocholanolone), 82 ± 1.5% (epiandrosterone), 93.7 ± 1.7% (17-hydroxy-pregnenolone), 91.8 ± 1.9% (dehydro-epiandrosterone), 77.4 ± 1.5% (estrone), 79.6 ± 2.3% (pregnenolone), 92.5 ± 2.8% (epipregnanolone, 3βOH-5β-pregnane-20-one), 84 ± 2.2% (allopregnanolone), 66.8 ± 2% (pregnanolone), and 72.4 ± 2% (isoallopregnanolone) (mean ± SEM). The analysis methods have been described earlier [19].

These values indicate that the estimation of the steroid sulfate/glucuronides using this hydrolysis method, suggested by Dehennin et al., reflects the actual circulating levels of the sulfate/glucuronided steroids [69]. It would be appropriate to use the obtained values for correction of the results, but as sulfates were commercially available only for a minority of the steroids investigated, this was not done. The losses of steroids, which were independent of the corresponding hydrolysis step, were covered by the addition of an internal standard into the polar residue after the diethyl-ether extraction.

### 4.5. Data Work up

To be able to compare the amount of the different metabolites formed after treatment with the two different steroids, we calculated adjusted concentration changes of the analyzed steroids. This was done by dividing the change in metabolite concentration for each tissue with the amount of steroid given. That is, for the allo-treated group, [metabolite]_treated_ − [metabolite]_control_/2 mg allo given/kg; for the isoallo-treated group, [metabolite]_treated_ − [metabolite]_control_/32 mg isoallo given/kg.

### 4.6. Statistical Analysis

Only non-parametric statistical tests were used. Within an animal group, comparisons were made with Friedman’s test, followed by the Mann–Whitney U-test when the original test was significant, i.e., comparisons between the adjusted concentrations of one steroid between the different tissues.

The adjusted changes of metabolites formed after allo and isoallo treatments were compared with the Mann–Whitney U-test and Bonferroni adjustment.

Between the three animal groups, the concentrations of the steroids were compared with the Kruskal–Wallis test, and with *p* ≤ 0.05, the Mann–Whitney U-test with Bonferroni adjustment identified which parameters differed. All values referring to steroid concentrations and adjusted changes are shown as median and upper and lower quartiles. In analyses regarding steroid concentrations in the striatum, one animal in the isoallo 32 mg/kg group was excluded due to problems during analysis. The SPSS version 28, statistical package (IBM Corp., Armonk, NY, USA) was used for statistical analysis.

## 5. Conclusions

In conclusion, both allopregnanolone and isoallopregnanolone metabolites were found both inside and outside of the CNS. As a high level of allo was found after the isoallo injection, there might be a high converting capacity within the brain or a large and rapid uptake into the brain. This suggests that the interchange between the epimers is of a physiological nature. One speculation may be that the brain uses the conversion between allo and isoallo to regulate the neurosteroid action on GABAergic inhibition. If this is the case, dysregulation in the metabolism might cause symptoms and/or CNS disorders.

## Figures and Tables

**Table 1:** Effective masses and retention times for analyzed steroids (quantifier is underlined, detected steroids in plasma and/or tissues are in bold).

Steroid	Effective Mass				Retention Time (min)		
	*m*/*z* (Da)				Peak 1	Peak 2	σ
**17α-estradiol** (internal standard)	**231**	**285**	**416**	**---**	**8.16**	**---**	**0.02**
3β-OH-5β-pregnane-20-one	241	298	388	---	9.79	---	0.03
**Allopregnanolone (allo)**	**241**	**298**	**388**	**---**	**10.18**	**---**	**0.03**
**7α-OH-allo**	**358**	**386**	**476**	**---**	**10.51**	**---**	**0.03**
3α-OH-5β-pregnane-20-one	241	298	388	---	10.52	---	0.03
**3β,5β-THDOC**	**282**	**386**	**476**	**---**	**12.14**	**---**	**0.02**
**Isoallo**	**241**	**298**	**388**		**12.25**	**---**	**0.02**
**3α5α-THDOC**	**476**	**---**	**---**	**---**	**12.34**	**---**	**0.02**
3α5β-THDOC	282	358	476	---	13.15	---	0.02
**6α-OH-isoallo**	**282**	**358**	**386**	**476**	**13.25**	**---**	**0.01**
**3β5α-THDOC**	**358**	**386**	**476**	**---**	**13.55**	**---**	**0.01**
5β-DHP	275	288	343	---	13.69	13.74	0.01
**5α-DHP**	**275**	**288**	**343**	**---**	**14.15**	**14.18**	**0.01**
5β-DHDOC	275	288	359	431	14.29	14.32	0.01
**5α-DHDOC**	**431**	**---**	**---**	**---**	**14.79**	**---**	**0.02**

**Table 2:** Circulating levels (nmol/L) of free and sulfate/glucuronided allo, isoallo, and some of their metabolites in male Wistar rat (shown as median with quartiles).

Steroid	Vehicle Group	Allo Treated Group	Isoallo Treated Group
Allo	28.5 (17.2, 34.5)	717 (635, 742) *	1080 (818, 1130) *
Isoallo	0.108 (0.096, 0.364)	1.68 (1.05, 1.98) *	24,700 (23,200, 31,000) *
5α-DHP	1.72 (1.53, 2.3)	35 (29, 38.1) *	292 (223, 317) *
5α-DHDOC	0.247 (0.176, 0.412)	0.473 (0.366, 0.568)	6.69 (4.13, 7.2) *
3α5α-THDOC	0.342 (0.244, 0.617)	2.26 (2.05, 2.88) *	136 (58.7, 177) *
3β5α-THDOC	0.044 (0.04, 0.638)	0.084 (0.065, 0.154)	179 (105, 218) *
7α-OH-allo	0.053 (0.039, 0.073)	0.221 (0.211, 0.232) *	98.8 (64.9, 105) *
6α-OH-isoallo	0.055 (0.036, 1.02)	0.673 (0.576, 0.993)	811 (478, 834) *
Allo-C	37.5 (12.5, 93.9)	795 (701, 1160) *	890 (495, 1090) *
Isoallo-C	5.09 (0.506, 17.1)	4.15 (2.5, 11.9)	19,300 (34,900, 48,200) *
5α-DHDOC-C	17.2 (12.1, 59.7)	10.3 (7.25, 31)	7.71 (1.86, 4.76) *
3α5α-THDOC-C	ND	ND	ND
3β5α-THDOC-C	ND	ND	ND
7α-OHallo-C	ND	ND	ND
6α-OH-isoallo-C	0.598 (0.192, 7.33)	0.981 (0.66, 2.31)	465 (203, 297) *

* *p* < 0.05 With Mann–Whitney U-test and Bonferroni correction.

**Table 3:** Levels of allo, isoallo, and some of their metabolites (nmol/kg) in the hippocampus of male Wistar rat (shown as median with quartiles).

Steroid	Vehicle Group	Allo Treated Group	Isoallo Treated Group
Allo	69 (33.7, 110)	2990 (2230, 3010) *	596 (590, 666) *
Isoallo	9.61 (4.41, 16.5)	54.2 (47.5, 60.8) *	36,300 (35,700, 37,000)
5α-DHP	4.62 (1.8, 7.07)	85.1 (55, 156) *	69.7 (33.4, 90.2) *
5α-DHDOC	12.5 (8.05, 17.6)	6.56 (5.45, 13.9)	3.12 (3.03, 4.53) *
3α5α-THDOC	0.374 (0.116, 1.26)	1.19 (0.617, 1.24)	8.99 (8.24, 11.2) *
3β5α-THDOC	0.963 (0.515, 2.39)	0.335 (0.16, 0.841)	10.8 (10.4, 13.8) *
7α-OHallo	ND	ND	42.4 (42.2, 57.3) *
6α-OH-isoallo	0.547 (0.06, 13.1)	0.549 (0.001, 11)	274 (210, 292) *

* *p* < 0.05 With Mann–Whitney U-test and Bonferroni correction.

**Table 4:** Levels of allo, isoallo, and some of their metabolites (nmol/kg) in the striatum of male Wistar rat (shown as median with quartiles).

Steroid	Vehicle Group	Allo Treated Group	Isoallo # Treated Group
Allo	40.4 (14.2, 60.9)	2010 (1690, 2450) *	611 (413, 731) *
Isoallo	8.43 (5.08, 67.3)	27.5 (21.7, 29.7)	45,300 (39,500, 53,400) *
5α-DHP	1.76 (0.784, 16.8)	37.3 (20.3, 49) *	116 (90.9, 129) *
5α-DHDOC	6.49 (4.81, 10.2)	4.15 (3.29, 8.77)	2.37 (2.08, 2.69) *
3α5α-THDOC	0.553 (0.258, 0.636)	0.855 (0.485, 1.46)	14.2 (10.4, 17.9) *
3β5α-THDOC	0.975 (0.503, 1.15)	0.354 (0.28, 0.652)	30.1 (28.7, 32.3) *
7α-OHallo	ND	ND	29.2 (6.28, 35.1)
6α-OH-isoallo	0.247 (0.181, 35.5)	0.893 (0.756, 1.54)	194 (170, 246) *

* *p* < 0.05 With Mann–Whitney U-test and Bonferroni correction, # n = 4, significant change without Bonferroni correction.

## Data Availability

The original contributions presented in this study are included in the article. Further inquiries can be directed to the corresponding author.
